# Immunotherapy-Induced Glutamic Acid Decarboxylase 65 Cerebellitis

**DOI:** 10.7759/cureus.109334

**Published:** 2026-05-21

**Authors:** Alysse Jensen, Hardik Thakkar, Scott Kramer, Jacqueline Young

**Affiliations:** 1 Internal Medicine, Moffitt Cancer Center, Tampa, USA

**Keywords:** gad 65, glutamic acid decarboxylase antibodies (gad-65), ici cerebellitis, ici neuro, immune check point inhibitor adverse effects, immune checkpoint inhibitor adverse effects, immune checkpoint inhibitors (icis), neuro-oncology, oncology

## Abstract

Immune checkpoint inhibitors (ICIs) are an important treatment modality for advanced cancer. However, they carry the risk of immune-related adverse events (irAEs) secondary to their mechanism of action. ICI medications function by inhibiting the checkpoints that suppress immune cells, which exist to prevent autoimmunity, and prime these cells to attack and eliminate cancer. While ICIs have substantially improved the prognosis for patients with advanced cancer, some patients experience adverse effects from this autoimmune state. One less common, but potentially life-threatening toxicity is cerebellitis.

We present a case of anti-glutamic acid decarboxylase 65 (anti-GAD 65) related ICI cerebellitis in a patient with uterine carcinosarcoma on dostarlimab. This patient presented with nonspecific symptoms, including fatigue and a concerning symptom of ataxia. Full neurological evaluation demonstrated anti-GAD 65 antibodies in both her serum and cerebrospinal fluid (CSF). She was unable to start outpatient treatment and was admitted for intravenous steroids and intravenous immunoglobulin (IVIG) with subsequent improvement in her symptoms. She was placed on a prolonged steroid taper with regular IVIG infusions. Her immunotherapy was discontinued. This case report highlights the importance of early clinical suspicion of ICI-related neurotoxicities and rapid treatment initiation. Prompt recognition and early intervention are important to minimize long-term disability.

## Introduction

There are three classes of immune checkpoint inhibitors (ICIs), each targeting a different cell surface protein. These classes include programmed cell death receptor 1 (PD-1), programmed death ligand 1 (PD-L1), and cytotoxic T-lymphocyte-associated antigen 4 (CTLA-4) [1]. There are a multitude of malignancies that are treated with ICI therapy, improving long-term outcomes [2]. However, there are notable adverse effects from ICI treatment due to their mechanism of action. ICIs function to prime immune cells to attack cancer cells via the removal of ICIs, which places patients in an effective state of autoimmunity [1]. This autoimmunity can result in adverse effects that range from fatigue or rash to acute cardiac or neurologic toxicities. Neurological immune-related adverse events (irAEs) are both diverse and uncommon, and their presentations depend on the anatomic location affected. Guidon et al. broadly divide neurological irAEs into central nervous system (CNS) or peripheral nervous system (PNS) manifestations [3]. CNS manifestations include meningitis, encephalitis (including cerebellitis and limbic encephalitis), demyelinating syndromes, and vasculitides. PNS manifestations include neuropathies, neuromuscular junction disorders (such as myasthenia gravis), and myopathies. Neurological irAEs are rare and can be life-threatening if not addressed in a timely manner [4-6]. Here we present a case of a 63-year-old patient with ICI cerebellitis secondary to her PD-1 inhibitor (dostarlimab) maintenance therapy.

## Case presentation

A 63-year-old female patient presented with a history of stage IIIB2 uterine carcinosarcoma status post robotic hysterectomy and bilateral salpingo-oophorectomy in 2024 and six cycles of carboplatin, paclitaxel, and dostarlimab. She continued maintenance dostarlimab for 10 months prior to her presentation. Additional past medical history included left cavernous sinus meningioma status post resection with left cranial nerve (CN) VI paresis, deep vein thrombosis, depression, chronic low back pain, and chemotherapy-induced peripheral neuropathy. She presented due to two months of worsening fatigue, generalized weakness, lightheadedness, and ataxia, manifesting as gait instability. She was admitted for a full neurological evaluation.

Vital signs were unremarkable, except for an elevated blood pressure of 156/85 mmHg. She was otherwise afebrile, without tachycardia, and oxygen saturation was 98% on room air. Physical examination revealed the patient was alert and oriented to herself, her location, and the month, day, and year. She was able to follow all commands with her comprehension and attention intact. She was without dysarthria and aphasia. All cranial nerves were intact, but with chronic left CN VI palsy. Pupils were equal and reactive to light, full quadrants, intact extraocular muscles, facial movements intact, intact V1/V2/V3 sensation, hearing intact, tongue midline, and with good strength with bilateral side movements. All extremities moved equally with 5/5 strength. Sensory loss distal to the midshins, stated that pinprick sensation drops to 70% distal to one-third of the bilateral shins. Romberg positive. Deep tendon reflexes were 2+ and symmetric bilaterally in the biceps, triceps, brachioradialis, patellar, and Achilles tendons. Negative Hoffmann's sign bilaterally. Neck non-stiff, non-tender with negative Brudzinski and Kernig's signs. No finger-to-nose or heel-to-shin dysmetria. Gait was ataxic with short steps.

Laboratory tests included a complete blood count (CBC), comprehensive metabolic panel (CMP), erythrocyte sedimentation rate (ESR), C-reactive protein (CRP), creatine kinase (CK), aldolase, lactic acid, procalcitonin, antinuclear antibody (ANA), copper, ammonia, vitamin B12, thyroid stimulating hormone (TSH), cortisol, and adrenocorticotropic hormone (ACTH) to further evaluate for infectious, inflammatory, and endocrine causes of the patient’s dizziness. The ESR and lactic acid were elevated to 30 mm/hour (reference range 0-20 mm/hour) and 2.2 mmol/L (reference range 0.5-2.0 mmol/L), respectively. All other lab tests were unremarkable, including the white blood cell count. Central nervous system radiographic imaging included computed tomography (CT) of the head and neck, CT angiogram (CTA) of the head and neck, and magnetic resonance imaging (MRI) of the brain, all of which did not demonstrate any acute abnormalities (Figures 1-3). Additionally, a carotid doppler found no hemodynamically significant stenosis, and an MRI of the total spine found no evidence of significant spinal canal compromise in the cervical, thoracic, and lumbar spine, as well as no spinal metastasis. A lumbar puncture (LP) was notable for lymphocytic pleocytosis and oligoclonal bands, with the opening pressure, total protein, and glucose within normal limits (Table 1). Additional laboratory analyses of serum and cerebrospinal fluid (CSF) were obtained and sent for further testing to an outside laboratory. The patient was discharged with close neuro-oncology follow-up.

**Figure 1:**
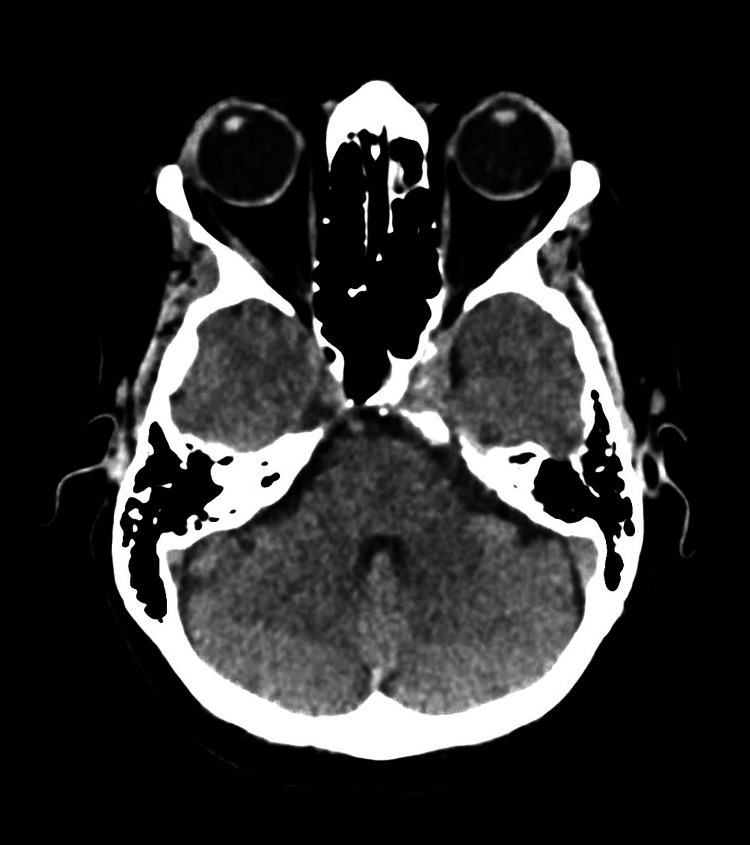
Non-contrast CT of the head (axial cut), demonstrates no acute intracranial abnormality. This negative finding helped exclude structural causes of ataxia. Negative findings on a CT head do not rule out immune checkpoint inhibitor cerebellitis. CT: computed tomography

**Figure 2:**
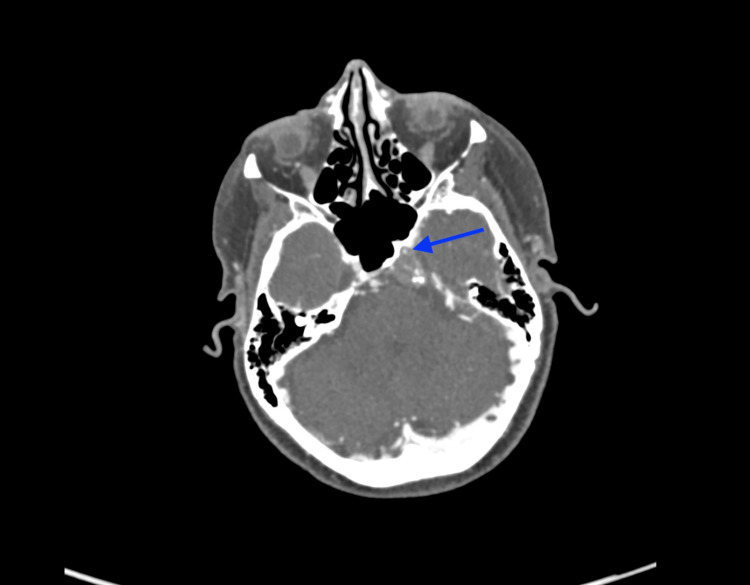
CTA of the head and neck (axial cut). Presumed meningioma involving the left cavernous sinus creates a 75% narrowing of the left cavernous sinus artery (arrow). No significant stenosis, flow limiting lesion, or occlusion of the cervical arterial tree are noted. CTA: computed tomography angiography

**Figure 3:**
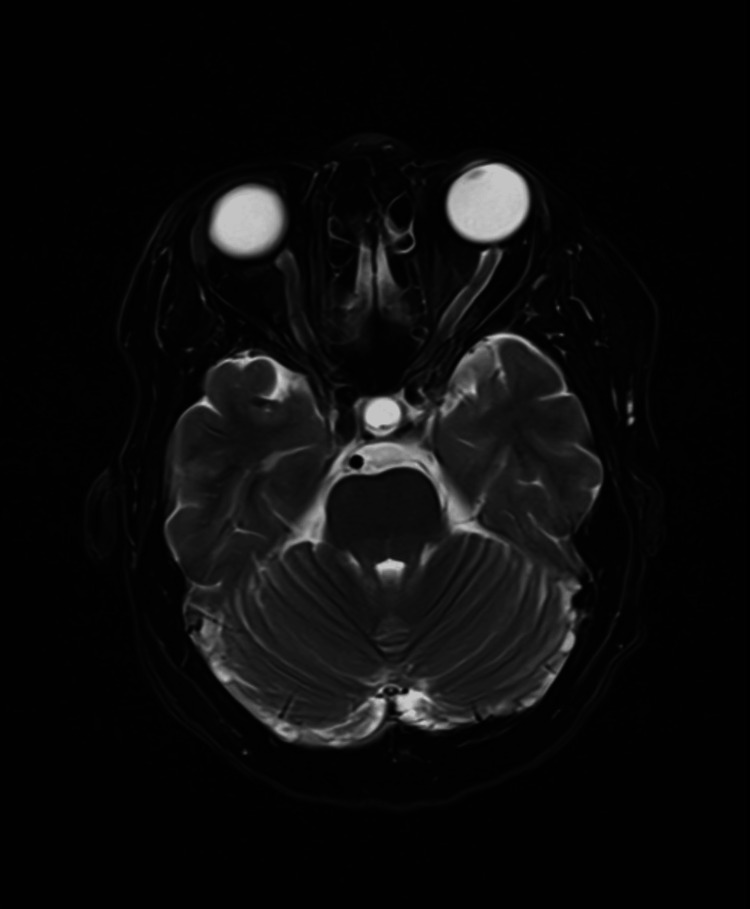
Contrast-enhanced T2-weighted MRI shows no cerebellar high-signal intensity. There are no acute findings to explain the patient's dizziness, and there is no change compared to the prior study. The observed contrast enhancement is negative, but this does not rule out immune checkpoint inhibitor cerebellitis. MRI: magnetic resonance imaging

**Table 1:** Lumbar puncture results and serum GAD 65 levels. CSF: cerebrospinal fluid; GAD 65 Ab: glutamic acid decarboxylase 65 antibody

Test	Value	Reference Range
Opening pressure	19 cm H20	10-20 cm H_2_O
Total nucleated cells (CSF)	7 cells (96% lymphocytes)	0-5 cells
Protein (CSF)	45 mg/dL	15-45 mg/dL
Glucose (CSF)	55 mg/dL	40-70 mg/dL
Gram stain (CSF)	No growth	No growth
Oligoclonal bands (CSF)	Positive, 7 bands	Negative
GAD 65 Ab assay (CSF)	35.4 nmol/L	≤ 0.02 nmol/L
GAD 65 Ab (serum)	> 250 IU/mL	0-5 IU/mL

At the neuro-oncology office, the patient reported worsening symptoms. The CSF and serum outside lab tests had finally resulted weeks after sending to the laboratory and were positive for anti-GAD 65 antibodies in both the serum and CSF, with marked elevations of 50 and 1,770 times the upper limit of normal, respectively (Table 1). Given her presentation and anti-GAD 65 antibodies, she was diagnosed with ICI cerebellitis, a subtype of ICI encephalitis. She was prescribed an outpatient prednisone taper and intravenous immunoglobulin (IVIG), but given the severity of her symptoms, she was unable to start this regimen and was instead re-admitted for treatment. She received three days of intravenous methylprednisolone at 1g/day and two days of IVIG at 1g/kg. She had rapid improvement in her symptoms, with almost complete resolution of her ataxia, and was discharged on a prolonged steroid taper with regular IVIG infusions scheduled. Her maintenance dostarlimab was discontinued, and she began surveillance of her uterine cancer.

## Discussion

ICIs are an important treatment modality for advanced cancer, but are not without potential life-threatening adverse effects. The pathogenesis of these adverse effects is secondary to the mechanism of action that results in immune cells primed to attack and eliminate cancer, placing a patient in a potential state of autoimmunity. Encephalitis is an uncommon adverse effect but can result in significant morbidity and mortality [4].

The diagnosis of ICI encephalitis can be challenging, as there are numerous subtypes. Other conditions, including but not limited to infection, cerebrovascular events, and brain metastases, must be eliminated from the differential diagnosis. As in this case, vitals, laboratory tests, and an LP did not demonstrate an infectious etiology, electrolyte disturbance, vitamin deficiency, or positive ANA. The patient remained afebrile, without leukocytosis, and with no bacterial growth on the LP Gram stain. CNS imaging showed no evidence of a cerebrovascular event, new or worsening brain metastases, or leptomeningeal disease.

A systematic review and meta-analysis found that the median length of time on anti-PD-1 immunotherapy prior to adverse events is 40 days [4], whereas other studies found a median of 56-62 days [5, 6] regardless of immunotherapy class. The subtypes of ICI encephalitis include meningoencephalitis, limbic encephalitis, and cerebellitis. Most patients with ICI encephalitis present with fever, altered mental status, and/or memory changes [6]. ICI-related encephalitis is defined using the same criteria as autoimmune encephalitis (Table 2) [3]. First, a patient must present with signs and symptoms consistent with encephalitis and inflammation demonstrated by a brain tissue biopsy or improvement in symptoms following immunomodulatory therapy. CNS inflammation must be evidenced by MRI, CSF, or electroencephalogram (EEG) abnormalities or neural autoantibodies. Treatment for ICI toxicity includes steroids, IVIG, and other immunosuppressive medications such as infliximab, mycophenolate, and rituximab [5, 6].

**Table 2:** Immune checkpoint inhibitor (ICI) encephalitis diagnostic criteria. Adapted from Guidon et al. [3]. MRI: magnetic resonance imaging; CSF: cerebrospinal fluid; EEG: electroencephalogram

ICI Encephalitis Diagnostic Criteria	
Must meet criterion 1 and either 2a or 2b	
1	Signs and symptoms of encephalitis
2a	Brain biopsy with inflammation
2b	Central nervous system inflammation demonstrated by one of the following criteria and improvement following administration of steroids or immunomodulatory therapy: MRI with parenchymal abnormality, inflammation present in the CSF, abnormal EEG, and/or neural-specific autoantibodies in the serum and/or CSF

In the case presented above, the patient’s primary complaint was gait instability, as compared to the typical presentations of fever, altered mental status, and memory changes. At the time of presentation, she had received dostarlimab for a total of 15 months, which is an extended period on ICI therapy before an adverse event, compared to the median of 40-60 days. Additionally, this patient had no acutely abnormal findings on MRI or CT. An EEG was not performed during her admission, which may have been a limitation in ruling out seizure activity. However, CSF studies showed pleocytosis and oligoclonal bands, which can be associated with an inflammatory process within this patient’s CNS (Table 1). Furthermore, this patient’s CSF and serum levels of the neural autoantibody, GAD 65, were markedly elevated (Table 1). While these neural autoantibodies can be present in paraneoplastic neurologic disorders and autoimmune neurologic disorders, they have also been observed in cases of ICI encephalitis with a good prognosis [5]. Ultimately, neural autoantibodies can be useful in diagnosing ICI encephalitis, but their lack of specificity must be acknowledged, as discussed by Guidon et al. and Velasco et al. [3, 5]. This patient had exposure to an ICI, positive markers of inflammation in her CSF and serum, GAD 65 autoantibodies, and a relatively rapid response to immunomodulatory treatment and return to her baseline, consistent with the diagnosis of ICI cerebellitis.

It is difficult to quantify the number of ICI encephalitis cases per year, as there is limited data beyond small case reviews and systematic analyses [4-6]. In the gynecologic oncology field, the use of ICI therapy occurred later compared to other oncology fields, perhaps resulting in both fewer cases of neurological irAEs and reduced familiarity with their various presentations and subsequent management [7]. Across two systematic reviews and one retrospective series, a total of 31 patients expired from ICI neurologic adverse effects. Given this relatively uncommon but life-threatening toxicity, it is vital for healthcare providers in an oncology and internal medicine setting to be familiar with the signs, symptoms, diagnostic evaluation, and treatment algorithm. This case highlights the diversity in symptom presentation and the importance of rapid recognition and treatment initiation in patients currently on ICIs.

## Conclusions

The case presented above is unique in multiple aspects, such as the patient’s presentation of fatigue and ataxia, extended length of time on immunotherapy prior to an adverse event, lack of CNS imaging findings, GAD 65 antibodies present in the serum and CSF, and recovery to the patient’s baseline. As discussed previously, most cases of ICI encephalitis present as fever, altered mental status, and memory changes, with one-third to half of patients testing positive for autoantibodies. Additionally, most patients experience neurological irAEs within 40-60 days of therapy initiation and demonstrate abnormal findings on CNS imaging, unlike the case presented here. This case highlights the diverse presentation of ICI encephalitis and the importance of rapid identification and treatment for the potentially life-threatening irAE. This case is especially relevant given the rising use of ICI inhibitor therapy for the treatment of various malignancies and the increased risk of morbidity and mortality in neurological ICI toxicities.
